# Circulating T Cells of Patients with Nijmegen Breakage Syndrome Show Signs of Senescence

**DOI:** 10.1007/s10875-016-0363-5

**Published:** 2016-12-21

**Authors:** Ruud W. J. Meijers, Katarzyna Dzierzanowska-Fangrat, Magdalena Zborowska, Iwona Solarska, Dennis Tielemans, Bob A. C. van Turnhout, Gertjan Driessen, Mirjam van der Burg, Jacques J. M. van Dongen, Krystyna H. Chrzanowska, Anton W. Langerak

**Affiliations:** 1 Department of Immunology, Laboratory for Medical Immunology, Erasmus MC, University Medical Center Rotterdam, Wytemaweg 80, 3015 CN Rotterdam, The Netherlands; 2 Department of Clinical Microbiology and Immunology, The Children’s Memorial Health Institute, Warsaw, Poland; 3 Department of Medical Genetics, The Children’s Memorial Health Institute, Warsaw, Poland

**Keywords:** T cells, NBS, senescence, CD28null, sjTREC

## Abstract

**Purpose:**

The Nijmegen breakage syndrome (NBS) is an inherited genetic disorder characterized by a typical facial appearance, microcephaly, growth retardation, immunodeficiency, and a strong predisposition to malignancies, especially of lymphoid origin. NBS patients have a mutation in the *NBN* gene which involves the repair of DNA double-strand breaks (DSBs). Here we studied the peripheral T cell compartment of NBS patients with a focus on immunological senescence.

**Methods:**

The absolute numbers and frequencies of the different T cell subsets were determined in NBS patients from young age till adulthood and compared to age-matched healthy individuals (HI). In addition, we determined the expression of senescent T cell markers and the signal joint T cell receptor excision circles (sjTRECs) content.

**Results:**

Our results demonstrate that NBS patients have reduced T cell numbers. NBS patients showed lower numbers of αβ^+^ T cells, but normal γδ^+^ T cell numbers compared to HI. Concerning the αβ^+^ T cells, both CD4^+^ as well as CD8^+^ T cells were excessively reduced in numbers compared to aged-matched HI. In addition, NBS patients showed higher frequencies of the more differentiated T cells expressing the senescent cell marker CD57 and did not express co-stimulatory molecule CD28. These effects were already present in the youngest age group. Furthermore, NBS patients showed lower sjTREC content in their T cells possibly indicative of a lower thymic output.

**Conclusions:**

We conclude that circulating T cells from NBS patients show signs of a senescent phenotype which is already present from young age on and which might explain their T cell immune deficiency.

**Electronic supplementary material:**

The online version of this article (doi:10.1007/s10875-016-0363-5) contains supplementary material, which is available to authorized users.

## Introduction

The Nijmegen breakage syndrome (NBS) is an inherited autosomal recessive disorder which belongs to the group of chromosome instability syndromes. Characteristic for the disease is the facial appearance of patients in combination with microcephaly, growth retardation, an increased risk for malignancies, and immunodeficiency [1–3]. NBS patients have a mutation in the *NBN* gene (previously *NBS1*) located on chromosome 8q21. Over 90% of the patients contain a homozygous 5 nucleotide deletion (c.657del5) presumed to be of Slavic origin (founder effect), which causes premature termination at codon 219 [4].

By combining together with MRE11 and RAD50 in the so-called MRN complex, NBN is involved in the repair of DNA double-strand breaks (DSBs) [2, 5]. Such DSBs arise from ionizing radiation, oxidizing agents, but also occur in a physiological context during DNA replication, meiotic recombination, and V(D)J and class switch recombination [6]. The MRN complex acts as a marker of DNA breaks and is likely to be involved in both the homologous recombination (HR) and non-homologous end joining (NHEJ) pathways [7]. It is activated in response to DSBs, keeping the two DNA ends in close proximity [8]. Scanning force microscopy confirmed that juxtaposition of DNA ends indeed occurs through MRN interactions [9, 10]. The MRN complex has been shown to accumulate in large nuclear foci within minutes after DSB formation [11]. Collectively, this suggests that the MRN complex—probably in conjunction with other proteins—is involved in tethering the DNA ends to allow DNA repair proteins to complete their actions [12].

Another function of the MRN complex is to activate ATM, a crucial mediator of the cellular response to DNA damage. MRN recruits ATM to DNA breaks, which results in dissociation of the ATM dimer enabling ATM to become activated [13]. ATM phosphorylates many targets, including H2AX, involved in the regulation of cellular checkpoint response and DNA damage repair [14]. Phosphorylation of H2AX results in recruitment and retention of cell-cycle checkpoint proteins and DNA repair proteins. This allows to stop the cell-cycle and to initiate repair of DSBs [14]. Finally, the MRN complex plays a role in maintenance of chromosomal integrity in the cell [15].

Immune deficiency is a serious problem for NBS patients. Both defects in the development of lymphocytes but also in the lymphocytic function have been described [2, 16]. A mild to moderate lymphopenia contributes to a high risk for infections and malignancies in NBS patients [2, 17]. With increasing age, the absolute number of B cells remains invariably low [16].

Earlier, we showed a disturbed precursor B cell differentiation pattern and significant disturbances in the resolution of RAG-induced IGH breaks [2]. Despite this, gene usage and junctional region composition of the successful Ig gene rearrangements in NBS patients were highly similar to healthy controls [2]. This points to a quantitative rather than a qualitative defect in V(D)J recombination as caused by the NBN mutation. The result is a reduction in the bone marrow B cell efflux which appeared to be partly compensated by an increased proliferation of mature B cells [2, 16]. The inefficient V(D)J recombination in NBS patients is thought to eventually affect the broadness of the B cell receptor (BCR) repertoire, thus contributing to the observed immunodeficiency in NBS patients. Presumably, the loss of juxtaposed Ig genes will also lead to a higher risk of aberrant rearrangements, thus contributing to the increased risk of lymphoid malignancies in NBS patients.

In addition to the changes in the B cell compartment, decreased numbers of peripheral T cells were also observed [18]. The T cell compartment of NBS patients especially showed reduced numbers of naïve T cells [18]. Moreover, NBS patients had a relatively increased population of γδ^+^ T cell receptor (TCR) T cells and a reduced proportion of αβ^+^ TCR T cells.

Here, we further studied the peripheral T cell compartment of NBS patients with a focus on features that have been associated with immunological T cell senescence. Our data suggests a disturbed T cell system in NBS patients with clear signs of aberrant differentiation reflecting those of immunological senescence of T cells which is already apparent in the youngest NBS patient group.

## Methods

### Study Population

In this study, we included 20 NBS patients and grouped them based on their age (median, 4.6 (0.1–27.1) years old) into four groups and compared them with age-matched HI. One NBS patient (age, 1.3 years old) was cytomegalovirus (CMV)-seropositive based on the presence of anti-CMV IgG titers and viral DNA in blood. All patients carried the homozygous c.657del5 mutation in the *NBN* gene. In addition, peripheral blood samples of 171 HI were used (subdivided in four age cohorts: 0–2 years, *n* = 36; 2–5 years, *n* = 27; 5–16 years, *n* = 59; >16 years; *n* = 21). All obtained blood samples were collected according to the guidelines of the local Medical Ethics Committees of CMHI, Warsaw, PL, and Erasmus MC, Rotterdam, NL. The study was overall approved by the CMHI Institutional Review Board.

### Flow Cytometric Analysis of Peripheral T Cells

Absolute numbers of T (CD4^+^ and CD8^+^), B, and natural killer (NK) cells were determined by means of a BD TruCount^TM^ labeling (BD Biosciences, San Jose, Ca, USA). For this, 50 μl of peripheral blood was added to 20 μl BD multicolor antibodies containing CD3-fluorescein isothiocyanate (FITC), CD4-phycoerythrin (PE)-Cy7, CD16-PE, CD56-PE, CD8-allophycocyanin (APC)-H7, CD19-APC, and CD45-peridinin chlorophyll (PerCP) (Table 1) and incubated for 10 min at room temperature (RT). Next, 0.5 ml of NH_4_Cl was added followed by an incubation of 10 min at RT. Cells (minimum events of 100,000 lymphocytes) were measured a using FACSCanto II instrument (BD Biosciences).

**Table 1:** Six-color antibody panel for analyzing the composition of the T cell compartment

Tube	FITC	PE	PerCP	PE-Cy7	APC	APC-H7	
Tru Count	CD3 (SK7) ^A^	CD16/CD56 (B37.1)/(NCAM16.2) ^A^	CD45 (2D1) ^A^	CD4 (SK3) ^A^	CD19 (SJ25C1) ^A^	CD8 (SK1) ^A^	
1.	TCRαβ (WT31) ^A^	TCRγδ (11F2) ^A^	CD3 (SK7) ^A^	CD4 (SK3) ^A^	CD8 (SK1) ^A^		αβ vs. γδ
2.	CD45RO (UCHL1) ^B^	CD45RA (2H4) ^C^	CD3 (SK7) ^A^	CD4 (SK3) ^A^	CD27 (L128) ^A^	CD8 (SK1) ^A^	Maturation
3.	CD28 (CD28.2) ^A^	CD197 (3D12) ^A^	CD3 (SK7) ^A^	CD8 (SK1) ^A^	CD45RO (4CHL1) ^A^	CD27 (M-TL71) ^A^	Maturation
4.	CD57 (HNK-1) ^A^		CD3 (SK7) ^A^	CD4 (SK3) ^A^		CD8 (SK1) ^A^	Effector
5.	Vδ1 (R9.12) ^D^	Vδ2 (B6) ^A^	CD3 (SK7) ^A^	CD4 (SK3) ^A^	TCRαβ (IP-26) ^E^	CD8 (SK1) ^A^	γδ subset
6.	Vδ2 (B6.1) ^A^	Vγ9 (B3.1)^ A^	CD3 (SK7) ^A^	CD4 (SK3) ^A^	TCRαβ (IP-26) ^E^	CD8 (SK1) ^A^	γδ subset

Clones are shown between brackets, manufactures by characters: A—BD Biosciences, San Jose, CA, USA; B—Dako, Herverle, Belgium; C—Beckman Coulter, Woerden, the Netherlands; D—Immunotech, Marseille, France; E—eBioscience, San Diego, Ca, USA

In addition, the differentiation status of the T cell compartment was assessed. Approximately 2 ml blood was diluted in 50 ml NH_4_Cl followed by 10 min of incubation. After washing, cells were stained according to the six-color antibody labeling as shown in Table 1. To determine the different T cell subsets (tube 3 of table 1), T cells defined as CD3^+^ and either being CD8^+^ or CD8^−^ (CD4) were divided into different subsets on the basis of CD197 (CCR7) and CD45RO expression: naïve (CD197^+^CD45RO^−^CD27^+^CD28^+^), memory (CD197^+/−^CD45RO^+^CD27^+/−^CD28^+/−^), or effector (also known as effector memory CD45RA+ (EMRA); CD197^−^CD45RO^−^CD45RA^+^CD27^+/−^CD28^+/−^) T cells.

In addition, within the memory population, the frequency of central memory (CM; CD197^+^CD27^+^CD28^+^) and effector memory CD45RO^+^ (EMRO; CD197^−^CD27^+/−^CD28^+/−^) was determined. As markers for T cell senescence, frequencies of CD28null effector cells and CD57-expressing cells within the CD4^+^ and CD8^+^ population were determined.

Based on the TCR status, the frequency of αβ^+^ and γδ^+^ TCR within the CD3^+^ lymphocyte population was determined. In addition within the γδ^+^CD3^+^ T cells, we determined the usage of Vδ1 or Vδ2 chains [19].

### sjTREC Assay

For the δRec-ψJα signal joint (sj) TREC assay, DNA was isolated from isolated mononuclear cells (MNCs) using Sigma GenElute kits (Sigma Genosys, The Woodlands, TX, USA). Evaluation of the δRec-ψJα TRECs was performed by means of real-time quantitative (RQ-)PCR detection on the ABI Prism 7000 detection system (Applied Biosystems) and analyzed with SDS v.2.1 software from the same manufacturer. RQ-PCR was performed on 50 ng DNA in a 25 μl reaction mixture containing 700 nM of both forward primer (5′-TCGTGAGAACGGTGAATGAAG-3′) and reverse primer (5′-CCATGCTGACACCTCTGGTT-3′), 150 nM of hydrolysis probe 5′(FAM)-CACGGTGATGCATAGGCACCTGC-3′(TAMRA); and 12.5 μl 2× TaqMan Universal Master Mix (Applied Biosystems, Nieuwerkerk a/d IJssel, the Netherlands). The PCR protocol consisted of initial incubation at 50 °C for 2 min, denaturation at 95 °C for 10 min, followed by 45 cycles of denaturation at 95 °C for 15 s and annealing/elongation at 60 °C for 30 s. Quantification of the DNA amount in each sample was performed using an RQ-PCR of the single-copy albumin gene with the following primers: forward, 5′-TCGTGAGAACGGTGAATGAAG-3′; reverse, 5′-CCATGCTGACACCTCTGGTT-3′; probe, 5′(FAM)-CACGGTGATGCATAGGCACCTGC-3′(TAMRA). All reactions were performed in duplicate, whereas PCR experiments were repeated in case the ΔCT between replicates exceeded 1.5.

### Statistical Analysis

Comparison between NBS patients and HI and between the different age groups was done by using the one-way ANOVA parameter with the Kruskal-Wallis test which is the non-parametric variant, followed by the Dunn’s multiple comparisons as post-hoc test. The analysis of the sjTREC content was done by a *t* test followed by the non-parametric Mann-Whitney test which was used to determine differences between NBS patients and HI. For all analyses, *p* values <0.05 for two sides were considered statistically significant.

## Results

### NBS Patients Have a Decreased Number of Circulating B and T Lymphocytes

**Fig. 1:**
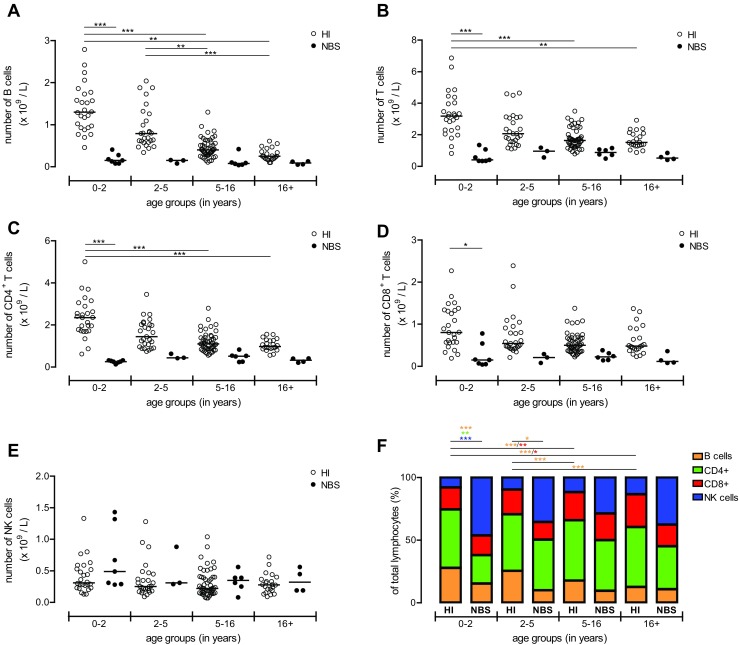
Absolute numbers of peripheral lymphocytes. The absolute number of lymphocytes was assessed by flow cytometry of healthy individuals (*HI*; *n* = 125, *open dots*) and of the NBS patients (*n* = 20, *black dots*). Patients and HI were divided on the basis of their age into four groups (age distribution (*n* = HI vs. NBS), respectively): 0–2 (*n* = 26 vs. 7), 2–5 (*n* = 27 vs. 3), 5–16 (*n* = 50 vs. 6), and 16+ years old (*n* = 22 vs. 3). The absolute number of B cells (**a**), total CD3^+^ T cells (**b**), CD4^+^ T cells (**c**), CD8^+^ T cells (**d**), and NK cells (**e**) was shown. Data represents individual measurements and medians. Significant differences between patients and HI and between different age groups are shown (* = *p* < 0.05, ** = *p* < 0.01, *** = *p* < 0.001). In addition within the total lymphocytes, the distribution of B cells (*orange bar*), CD4^+^ T cells (*green bar*), CD8^+^ T cells (*red bar*), and NK cells (*blue bar*) was shown (**f**). Data represents means and significant differences between patients and HI and between different age groups were shown (* = *p* < 0.05, ** = *p* < 0.01, *** = *p* < 0.001; the *color of the symbols* represents the different lymphocytes which were significantly different)

By using TruCount tubes, absolute number of T, B, and NK cells were determined from peripheral blood of NBS patients and compared with aged-matched HI (Fig. 1). Compared to HI, absolute numbers of B cells (Fig. 1a) and total T cells (Fig. 1b) were drastically reduced in peripheral blood of NBS patients [20]. This was especially true in the youngest age group (0–2 years). The absolute numbers of B and T cells for the older NBS patients are within normal range due to decreasing cell numbers for HI as the B- and T cell numbers remained low with increasing age for the NBS patients (Fig. 1a, b).

Further analysis of the T lymphocyte population revealed that both the CD4^+^ (Fig. 1c) and CD8^+^ (Fig. 1d) subsets showed this reduction with a slight normalization to the lower level of normal numbers in the older NBS patients. Interestingly, the absolute number of NK cells remained within the normal range in the vast majority of NBS patients (Fig. 1e).

When comparing frequencies of the different lymphocyte types between HI and NBS, it became clear that especially in the youngest age group the lymphocyte population in peripheral blood of NBS patients was composed of mainly NK cells (*p* < 0.001) and a lower frequency of especially CD4^+^ T cells (*p* < 0.01) and B cells (*p* < 0.001) (Fig. 1f). In the older age groups, the relative distribution of lymphocyte subsets slightly normalized.

### Lower αβ^+^ but Normal Numbers of γδ^+^ T Cells in NBS Patients

**Fig. 2:**
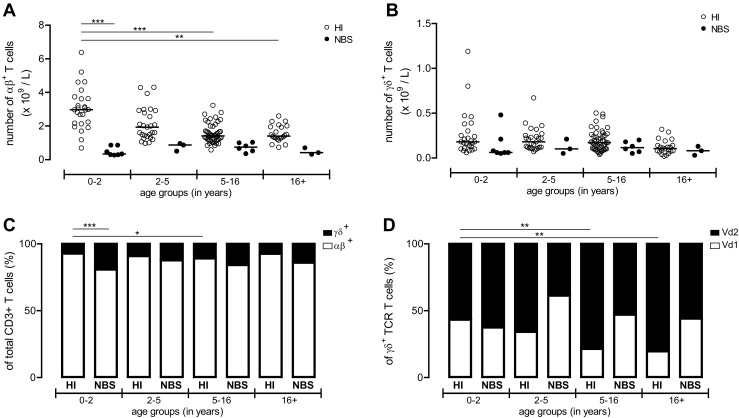
Absolute numbers and ratio of αβ and γδ T cells. Absolute numbers of αβ^+^ (**a**) and γδ^+^ (**b**) TCR T cells was determined in HI (*n* = 125, *open dots*) and in the NBS patients (*n* = 20, *black dots*). In addition, the distribution of αβ^+^ (*white bar*) and γδ^+^ (*black bar*) TCR T cells within CD3^+^ lymphocytes was shown (**c**) and the distribution of Vδ1^+^ (*white bar*) and Vδ2^+^ (*black bar*) TCR T cells within γδ^+^ TCR T cells was determined and shown (**d**). Both groups were divided on the basis of their age into four groups (age distribution (*n* = HI vs. NBS), respectively): 0–2 (*n* = 26 vs. 7), 2–5 (*n* = 27 vs. 3), 5–16 (*n* = 50 vs. 6), and 16+ years old (*n* = 22 vs. 3). Data represents individual measurements and medians in **a** and **b** and means in **d** and **e**. Significant differences between patients and HI and between different age groups were calculated and shown (* = *p* < 0.05, ** = *p* < 0.01, *** = *p* < 0.001)

The TCR of the majority of the T cell population was composed of TCR-αβ^+^ molecules but a small population of T cells (1–10%) contains a γδ^+^ TCR [21]. Compared to HI, NBS patients had significantly (*p* < 0.001) lower numbers of αβ^+^ TCR T cells (Fig. 2a) and normal numbers of γδ^+^ TCR T cells (Fig. 2b) in the youngest age group. Distribution of the CD3^+^ lympho-cytes into αβ^+^ and γδ^+^ TCR T cells showed that the youngest group of NBS patients has a significantly (*p* < 0.001) higher frequency of γδ^+^ TCR T cells due to lower numbers of αβ^+^ TCR T cells compared to age-matched HI (Fig. 2c) which is in line with earlier findings of Michalkiewicz et al. [18]. Numbers and frequencies of αβ^+^ TCR T cells in the older NBS patients were not different compared to the older HI (Fig. 2a, c).

In addition, absolute numbers of Vδ1^+^ (Supplementary Fig. S1A) and Vδ2^+^ (Supplementary Fig. S1B) γδ^+^ TCR were determined, but we did not detect differences between NBS patients and HI. Also the distribution of γδ^+^ TCR T cells into Vδ1 and Vδ2 γδ^+^ TCR frequencies did not show differences between NBS patients and HI in the matched age groups (Fig. 2d).

### Shift in Composition of NBS Peripheral T Cell Compartment Towards More Differentiated Cell Types

**Fig. 3:**
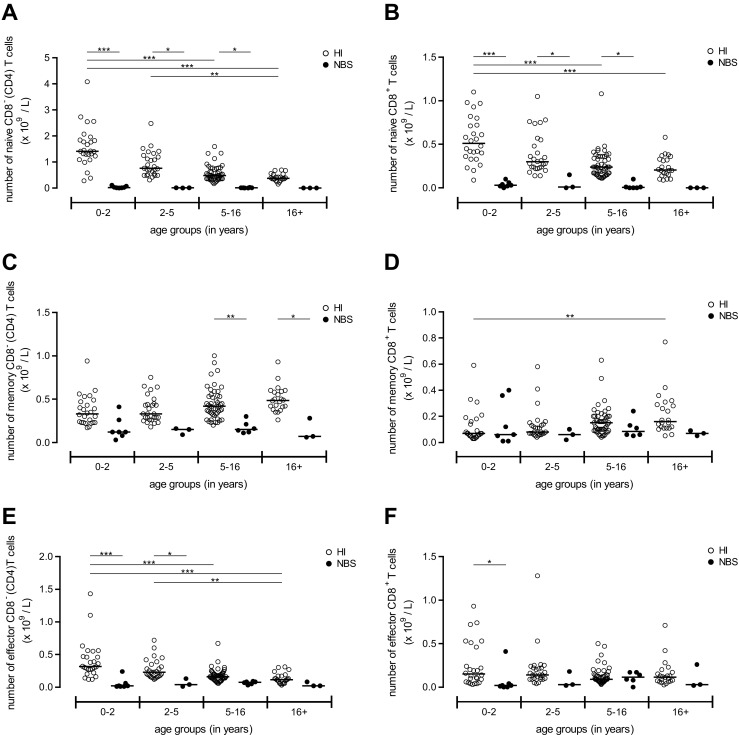
Absolute number of naïve, memory, and effector T cell subsets. Absolute numbers for the different T cell subsets were displayed for NBS patients (*n* = 20, *black dots*) which were compared to healthy individuals (*HI*) (*n* = 125, *open dots*). Both groups were divided on the basis of their age into four groups (age distribution (*n* = HI vs. NBS), respectively): 0–2 (*n* = 26 vs. 7), 2–5 (*n* = 27 vs. 3), 5–16 (*n* = 50 vs. 6), and 16+ years old (*n* = 22 vs. 3). First the absolute numbers of naïve T cells were shown for the CD8^−^ (CD4) (**a**) and CD8^+^ (**b**) T cell population. Second, the number of memory CD8^−^ (CD4) (**c**) and CD8^+^ (**d**) T cells was shown and third numbers of effector CD8^−^ (CD4) (**e**) and CD8^+^ (**f**) T cells. Data represents individual measurements and medians. Significant differences between patients and HI and between different age groups were calculated and shown (* = *p* < 0.05, ** = *p* < 0.01, *** = *p* < 0.001)

**Fig. 4:**
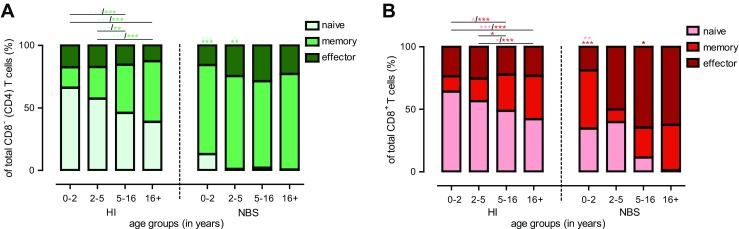
Distribution of the different T cell subsets. Within the CD8^−^ (CD4) (**a**; *green bars*) and CD8^+^ (**b**; *red bars*) T cell population, the distribution of the naïve (*lightest bar*), memory (*darker bar*), and effector (*darkest bar*) T cell subsets was shown for healthy individuals (*HI*) (*n* = 125) which were compared to NBS patients (*n* = 20). Both NBS as well as HI were divided on the basis of their age into four groups (age distribution (*n* = HI vs. NBS), respectively): 0–2 (*n* = 26 vs. 7), 2–5 (*n* = 27 vs. 3), 5–16 (*n* = 50 vs. 6), and 16+ years old (*n* = 22 vs. 3). Data represents means and significant differences between different age groups were calculated and shown. The symbols above the NBS bars represents significant differences between HI and NBS patients between same age groups (* = *p* < 0.05, ** = *p* < 0.01, *** = *p* < 0.001; the *color of the symbols* represents the different T cell subsets which were significantly different)

Next we examined the composition of the peripheral T cell compartment with respect to absolute numbers (Fig. 3) and relative distribution (Fig. 4 and Supplementary Fig. S3) of T cell subsets. Supplementary Fig. S2 represents flow cytometric examples of the gating strategy how the naïve, memory, and effector cells were defined in CD4^+^ and CD8^+^ T cells (Fig. S2A and C). Our healthy controls clearly show an age-dependent shift from naïve T cells towards memory T cells for both CD8^−^ (CD4) and CD8^+^ T cell populations as the absolute numbers (Fig. 3) and frequencies (Fig. 4) of naïve cells decline and the memory T cell compartment increases upon ageing.

By comparing absolute numbers of T cell subsets of NBS patients and HI, it became clear that NBS patients showed reduced numbers of naïve (Fig. 3a, b), memory (Fig. 3c, d), and effector cells (Fig. 3e, f) for both CD8^−^ (CD4) and CD8^+^ T cells, with most significant effects seen in the naïve and effector T cells.

However, when comparing the frequencies of the different T cell subsets within the total CD8^−^ (CD4) (Fig. 4a and S3A) and CD8^+^ (Fig. 4b and S3B) T cell population, percentages of naïve CD8^−^ (CD4) (Fig. 4a and S3A) and naïve CD8^+^ (Fig. 4b and S3B) T cells were significantly reduced for NBS patients as compared with HI at the youngest age. Notably, the frequency of naïve CD8^−^ (CD4) T cells was significantly reduced compared to age-matched HI in all age groups (Fig. 4a and S3A). In addition, the frequency of peripheral CD8^−^ (CD4) and CD8^+^ memory T cells (Fig. 4a, b and S3C and D) in NBS patients was significantly (*p* < 0.001) higher than in HI for the youngest group of patients. Interestingly, the memory T cell population appeared to be maximal at the youngest age without further increasing with increasing age in NBS patients (Fig. 4a, b and S3C and D).

The frequency of effector cells was not significantly increased in the very young NBS patients, but it appeared to gradually expand in NBS patients after 5 years especially for the CD8^+^ T cells (Fig. 4a, b and S3E and F).

Within the memory T cell population, frequencies of CM and effector memory CD45RO^+^ (EMRO) T cells were determined (Supplementary Fig. S3G-J). NBS patients had a significantly lower frequency of CM CD8^−^ (CD4) T cells as compared to HI, which was already apparent in the young age group and did not recover (Fig. S3G). The frequencies of CM CD8^+^ T cells were low and did not differ much from HI (Fig. S3H). Concerning the EMRO T cell subset, the frequency of EMRO CD8^−^ (CD4) T cells but not CD8^+^ T cells was significantly higher in the young age group for the NBS patients (*p* < 0.001) and the same trend was found for patients at higher ages (Fig. S3I and J).

Collectively, our results demonstrate that NBS patients have reduced numbers of naïve T cells for both CD8^−^ (CD4) and CD8^+^ T cells, and relatively expanded memory CD8^−^ (CD4) T cell and effector CD8^+^ T cell populations compared with age-matched HI. These alterations in T cell subsets are already present in the youngest age group and hardly change with increasing age.

### Increased Expression of Senescence Markers by T Cells in NBS Patients

As markers for highly differentiated or senescent T cells, we then assessed the frequency of CD57^+^ T cells and the frequency of effector (CD197^−^CD45RO^−^) T cells that lost the expression of co-stimulatory molecule CD28 [22, 23]. Supplementary Fig. S2B and D shows a representative example of the gating strategy. As confirmed in HI, with increasing age, frequencies of CD57^+^ T cells and CD28null T cells were increasing.

**Fig. 5:**
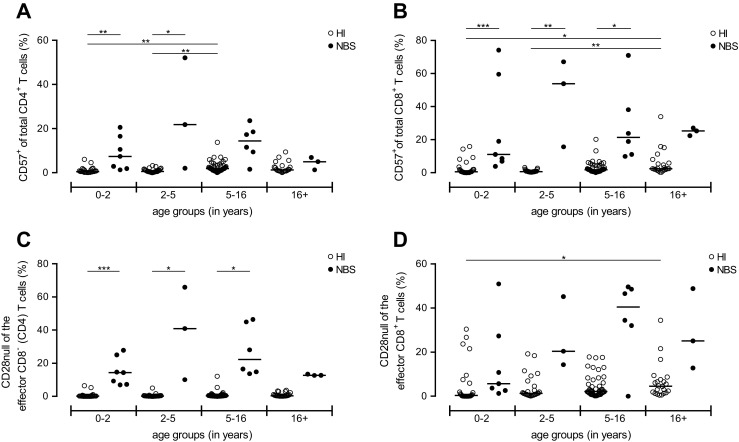
Frequency CD28null and CD57-expressing T cells. Frequencies of peripheral CD57^+^ cells within the CD4^+^ (**a**) and CD8^+^ (**b**) T cells were determined in both NBS patients (*n* = 20, *black dots*) and HI (*n* = 125, *open dots*). Next, the loss of co-stimulatory molecule CD28 within the effector fraction (CD45RO^−^CD197^−^) of the CD8^−^ (CD4) (**c**) and CD8^+^ (**d**) T cell population was determined. Both groups were divided on the basis of their age into four groups (age distribution (*n* = HI vs. NBS), respectively): 0–2 (*n* = 26 vs. 7), 2–5 (*n* = 27 vs. 3), 5–16 (*n* = 50 vs. 6), and 16+ years old (*n* = 22 vs. 3). Data represents individual measurements and medians. Significant differences between patients and HI and between different age groups were calculated and shown (* = *p* < 0.05, ** = *p* < 0.01, *** = *p* < 0.001)

The frequency of peripheral CD57^+^ T cells was determined within total CD4^+^ and CD8^+^ T cell population (Fig. 5a, b). NBS patients showed a clear increase in CD57-expressing cells within both CD4^+^ (Fig. 5a) and CD8^+^ T cells (Fig. 5b) compared to the HI.

In line with our findings for CD57, the same pattern was found for the frequency of CD28null T cells within the CD8^−^ (CD4) effector T cell population (Fig. 5c). The same increment was found in CD8^+^ effector T cells, although this did not reach statistical significance (Fig. 5d).

Taken together, these analyses show that NBS patients have a higher frequency of T cells with a senescent phenotype.

### NBS Patients Have a Lower sjTREC Content

**Fig. 6:**
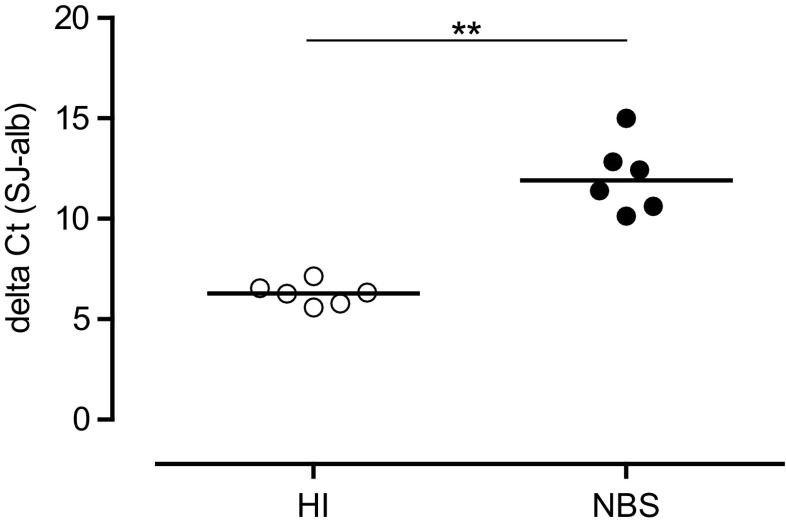
Output of naïve T cells by sjTREC content. The single joint T cell receptor excision circle (sjTREC) content was measured as a marker for thymic output of naïve T cells. The *y*-axis represents the ΔCt (difference in amplification cycles between TREC and albumin as a control for DNA input). The TREC content was determined of six NBS patients (*black dots*) and compared to eight age-matched HI (*open dots*). Data represents individual measurements and medians. Significant differences between patients and HI and between different age groups were calculated and shown (** = *p* < 0.01)

Finally, we determined the signal joint TCR excision circles (sjTRECs) as an indication for thymic output of naïve T cells [24]. A higher delta threshold (sjTREC CT subtracted by the CT value for albumin to correct for the DNA input) indicates a lower output of naïve T cells by the thymus. The sjTREC content was determined in a subset NBS patient population (*n* = 6; mean age, 9.5 years old (range, 1–23)) and compared to HI (*n* = 6; mean age, 6.7 years old (range, 1–11)) as shown in Fig. 6. The delta CT for the NBS patients was significantly (*p* = 0.022) higher than that of HI suggestive of a lower thymic function in NBS patients possibly in combination with an increased proliferation of peripheral T cells.

## Discussion

In the results of this study, we showed that immune senescence in combination with a lymphopenia of the T cell compartment contribute to the immunodeficiency of NBS patients. In accordance to existing literature [2, 16, 18], NBS patients had reduced numbers of circulating B and T lymphocytes. Concerning T cells, both CD4^+^ and CD8^+^ T cell numbers were reduced compared to age-matched HI. Upon division of the T cell population into subsets, our results clearly showed that for both CD8^−^ (CD4) and CD8^+^ T cells there was a shift towards the memory and effector compartment at the expense of the naïve compartment. Zooming into the memory T cells of NBS patients demonstrated that NBS patients had a higher frequency of senescent cells based on the loss of co-stimulatory molecule CD28 and the expression of CD57. Interestingly, most of the senescence-related effects on T cells, especially for the CD4^+^ T cells, were already present in the youngest NBS patient group.

In addition to the differentiation status of the T cells, our results demonstrated that the low number of naïve T cells might be the result of a lower thymic output of cells which is in line with the lower sjTREC content for NBS patients. However, since the sjTREC content was determined within the total MNC population, it should be emphasized that the lower sjTREC content could also partly result from increased proliferation of peripheral T cells. Ideally, the sjTREC content has to be studied in a purified naïve T cell population, but their numbers are too small in NBS patients to accurately address this.

The observed effects for T cells in our current study are in line with our earlier published B cell data from which we concluded that the reduction in the B cell efflux from the bone marrow appeared to be partly compensated by an increased proliferation of mature B cells [2]. In fact the increased frequency of highly differentiated T cells might compensate a lower output of naïve T cells by the thymus in order to maintain the T cell pool. Unfortunately, studies into disturbed precursor T cell differentiation in NBS patients are greatly hampered by the lack of available thymus material of these patients. Nevertheless, our findings on the peripheral T cell compartment could help explaining the T cell deficiency in NBS patients.

It remains speculative what the reason is for the reduced numbers of αβ^+^ TCR T cells while the γδ^+^ TCR T cell numbers are normal, which results in a relative increase of peripheral γδ^+^ TCR T cells in the youngest NBS patients as also shown by Michalkiewicz et al. [18]. Under healthy conditions, only 1–10% of the total peripheral T cell population expresses a TCR which is composed of γδ instead of αβ chains [19]. TCR γδ^+^ T cells appear not to require antigen processing and major histocompatibility complex (MHC) presentation to become activated, which places these cells between the innate and adaptive immune system [25]. The fact that the numbers of γδ^+^ TCR T cells are unaltered in contrast to the αβ^+^ TCR T cells might imply that there could be extrathymic expansion of γδ^+^ TCR T cells in NBS patients [26]. The reduced thymic function as demonstrated by the TREC content supports the theory that T cells in NBS patients might be generated independently from the thymus [27]. In addition, it was shown that NBS1 mutant mice have a reduction in thymus cellularity [27]. These observations all suggest that the contribution of the thymus in maintaining the T cell population is limited and that T cells could be formed by either an increased homeostatic proliferation or by extrathymic expansion instead. During T cell development in the thymus, first the *TCRD* and *TCRG* genes rearrange. When a functional γδ^+^ TCR is formed, no further gene rearrangement takes place and the cell remains a γδ^+^ TCR T cell [28]. NBS1 was predominantly found to co-localize in foci with the *TCRA* locus [29] which suggests that the TCRG/TCRD rearrangement would be less sensitive for a quantitative recombination defect than *TCRB/TCRA* rearrangements in NBS patients.

The NBN-related defect in the MRN complex leading to DSBs is likely to be the underlying cause of the reduced numbers of T cells in NBS patients [2]. Lymphopenia-induced proliferation might be a major driver of the described effects on peripheral T cells (low number of naïve T cells, increased frequency of senescent cells, and lower sjTREC content) in NBS patients. Such excessive proliferation of T cells would also lead to a reduction of the telomere length, which is characteristic for senescent T cells [30]. Due to preservation of the telomere length, these cells are protected from apoptosis. Elevated telomerase activity, increased expression of telomerase reverse transcriptase (hTERT) gene, and downregulation of negative hTERT regulators may be underlying mechanism of preventing the cell to go into apoptosis [31]. As the MRN complex is involved in maintaining chromosomal integrity, it has also been linked to maintaining the telomere length (for instance by recruiting telomerase) [32]. It was found in immortalized T cell lines from NBS patients that telomerase was upregulated after extensive proliferation of the cells [31]. Maintaining the telomere length might be an underlying mechanism causing T cells to go into a senescent state instead of going into apoptosis. Unfortunately due to limited patient material, we were unable to determine telomerase activity or the telomere length in this study but this might be of interest in future cases.

NBS patients have a high risk of developing malignancies, including B- and T cell-related malignancies which are associated with an increased mortality [17]. Especially non-Hodgkin lymphomas, both B- and T cell types are common [33]. Interestingly, an increased frequency of TCR loci (especially TRB, TRG) are found not to be properly ligated (data not shown), which is suggestive of aberrant TCR rearrangements and might be a risk factor for developing lymphomas. In addition, decreased immune surveillance due to the T cell lymphopenia might be linked to a high risk factor for malignancies. Monitoring patients for TCR trans-rearrangements may therefore serve as an additional marker to identify NBS patients at a high risk for lymphoma formation [1].

## Conclusion

In conclusion, the peripheral T cell compartment of NBS patients shows clear signs of senescence which is already visible in the youngest age group. Our results demonstrate that patients have reduced numbers of CD4^+^ as well as CD8^+^ T cells which might be associated with by a lower thymic output of naïve T cells. Furthermore, NBS patients have a higher frequency of memory and effector T cells and increased expression of CD57 and loss of CD28 expression on the cell surface. These findings might be an explanation for the immunodeficiency and perhaps also for the increased risk of lymphoid malignancies in NBS patients.

## Electronic Supplementary Material

Below is the link to the electronic supplementary material.

Supplementary Fig. S1 Absolute numbers of Vδ1^+^ and Vδ2^+^ γδ^+^ TCR cells. Here the absolute numbers of Vδ1^+^ (A) and Vδ2^+^ γδ^+^ (B) γδ^+^ TCR T cells was shown. Both HI (open dots) as well as HI (black dots) were divided on the basis of their age into four groups (age distribution (*n* = HI vs. NBS), respectively); 0–2 (*n* = 26 vs. 7), 2–5 (*n* = 27 vs. 3), 5–16 (*n* = 50 vs. 6), and 16+ years old (*n* = 22 vs. 3). Individual measurements and medians were shown. Significant differences between patients and HI and between different age groups were calculated and shown (* = *p* < 0.05, ** = *p* < 0.01, *** = *p* < 0.001) (PDF 387 kb)

Supplementary Fig. S2 Representative FACS analysis to determine T cell subsets and senescent T cells. Supplementary Fig. S2 shows representative examples of FACS plots to determine the different T cell subsets, CD57+ T cells, and CD28null effector T cells in HI (A and B) and in NBS patients (C and D). First, the gating strategy to determine the T cell subsets (from the maturation tube (tube 3) of Table 1) in which the CD8^+^ and CD8^−^ (CD4) were selected from the CD3^+^ lymphocyte population. Both CD8^−^ (CD4) and CD8^+^ T cells were divided into subsets using CD197 (CCR7) and CD45RO into a naïve (CD197^+^CD45RO^−^), memory (CD197^+/−^CD45RO^+^), and effector (CD197^−^CD45RO^−^) population. Furthermore, the memory population was divided in two subsets; the CD197^+^CD45RO^+^ subset from which the CM (CD28^+^CD27^+^) T cells were defined and the CD197^−^CD45RO^+^ subset from which the frequency of EMRO (CD28^+/−^CD27^+/−^) T cells was determined (A and C). Next in Fig. S2B and D, the gating strategy is shown how we determined the frequency of CD57^+^ T cells in total CD8^+^ and CD8^−^ (CD4) T cell populations and the loss of CD28 within the effector population (as gated in A and C). (PDF 544 kb)

Supplementary Fig. S3 Frequencies of the T cell subsets. Within CD8^−^ (CD4) and CD8^+^ percentages of naïve (A and B), memory (C and D), effector (E and F), CM (G and H), and EMRO (I and J) are shown of the NBS patients (*n* = 20, black dots) which were compared to HI (*n* = 125, open dots). NBS patients and HI were divided on the basis of their age into four groups (age distribution (*n* = HI vs. NBS), respectively): 0–2 (*n* = 26 vs. 7), 2–5 (*n* = 27 vs. 3), 5–16 (*n* = 50 vs. 6), and 16+ years old (*n* = 22 vs. 3). Data represents individual measurements and medians. Significant differences between patients and HI and different age groups were shown (* = *p* < 0.05, ** = *p* < 0.01, *** = *p* < 0.001). (PDF 607 kb)
