# Supplementary figures and images for: Circulating T Cells of Patients with Nijmegen Breakage Syndrome Show Signs of Senescence
Source: J Clin Immunol. 2016 Dec 21;37(2):133–42. doi: 10.1007/s10875-016-0363-5 (PMC5325864; doi:10.1007/s10875-016-0363-5)

## Supplementary Figure 1

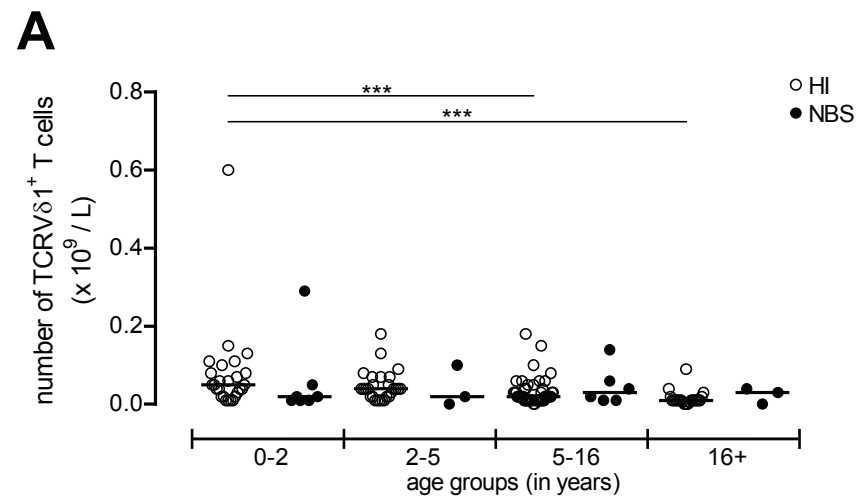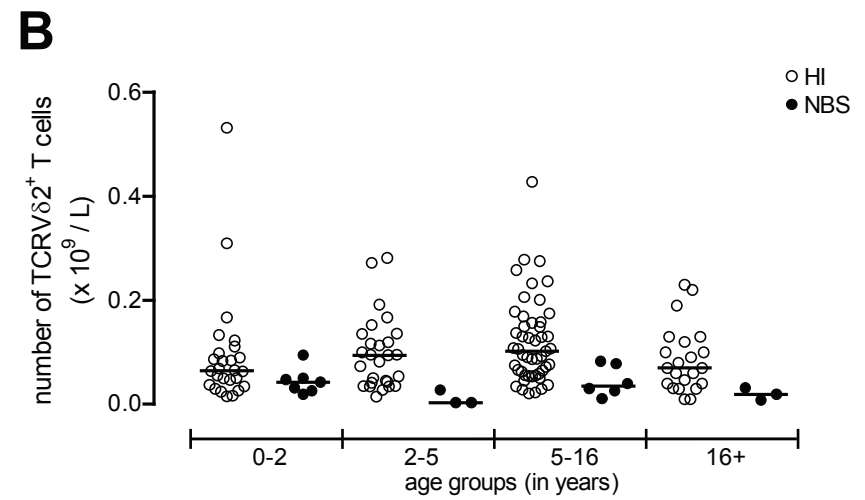

Supplement: Supplementary file 1 — Absolute numbers of Vδ1+ and Vδ2+ γδ+ TCR cells. Here the absolute numbers of Vδ1+ (A) and Vδ2+ γδ+ (B) γδ+ TCR T cells was shown. Both HI (open dots) as well as HI (black dots) were divided on the basis of their age into four groups (age distribution (n = HI vs. NBS), respectively); 0–2 (n = 26 vs. 7), 2–5 (n = 27 vs. 3), 5–16 (n = 50 vs. 6), and 16+ years old (n = 22 vs. 3). Individual measurements and medians were shown. Significant differences between patients and HI and between different age groups were calculated and shown (* = p < 0.05, ** = p < 0.01, *** = p < 0.001) (PDF 387 kb) [file 10875_2016_363_MOESM1_ESM.pdf]

# Supplementary Figure S2

**A**

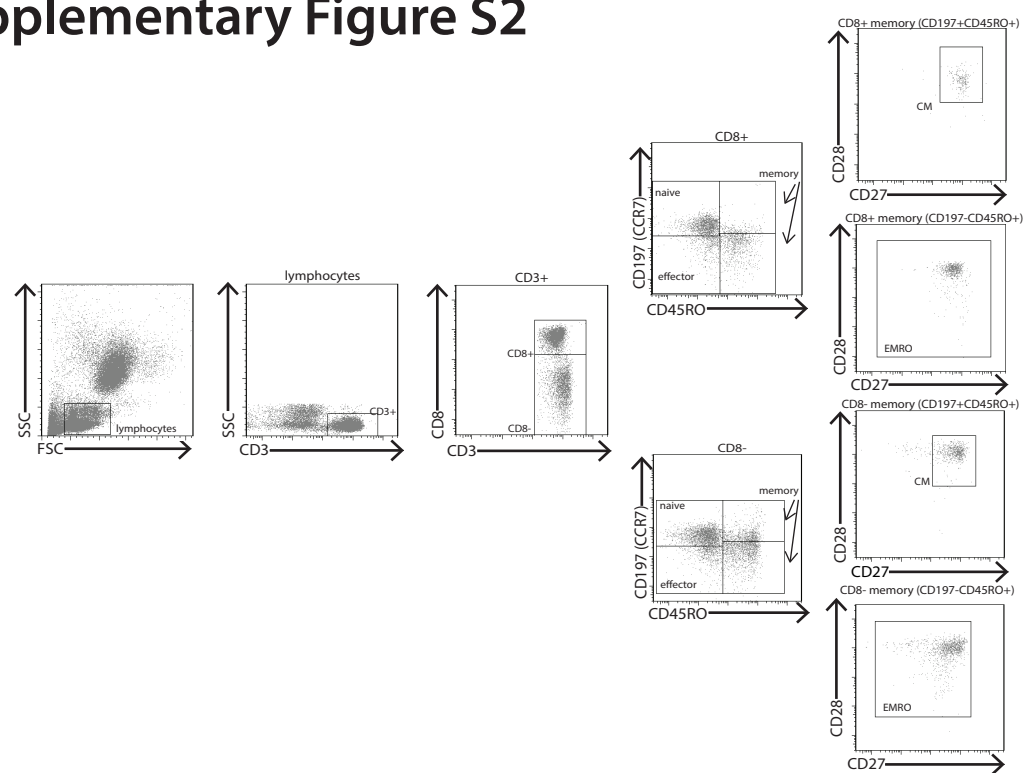

**B**

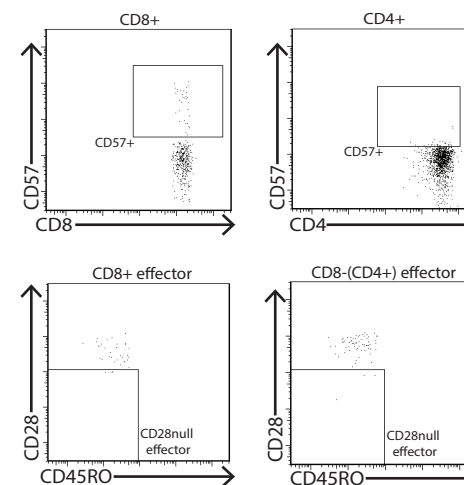

**C**

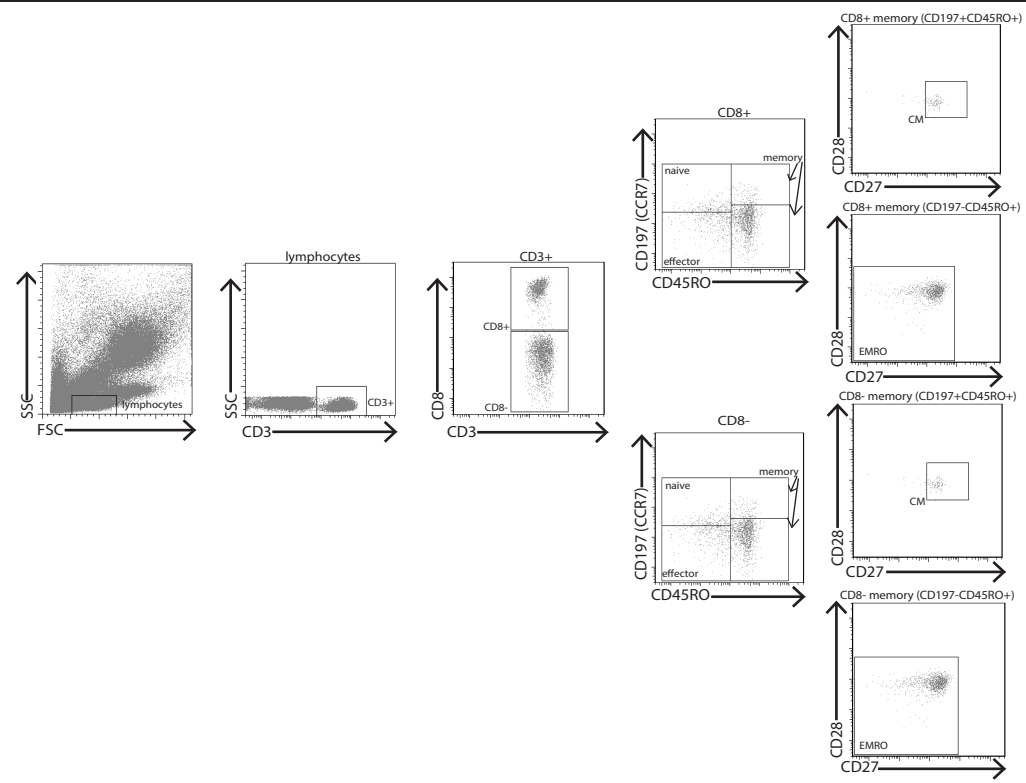

**D**

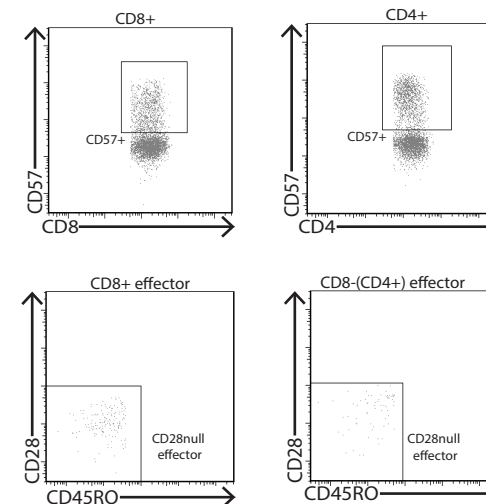

HI  
NBS

Supplement: Supplementary file 2 — Representative FACS analysis to determine T cell subsets and senescent T cells. Supplementary Fig. S2 shows representative examples of FACS plots to determine the different T cell subsets, CD57+ T cells, and CD28null effector T cells in HI (A and B) and in NBS patients (C and D). First, the gating strategy to determine the T cell subsets (from the maturation tube (tube 3) of Table 1) in which the CD8+ and CD8− (CD4) were selected from the CD3+ lymphocyte population. Both CD8− (CD4) and CD8+ T cells were divided into subsets using CD197 (CCR7) and CD45RO into a naïve (CD197+CD45RO−), memory (CD197+/−CD45RO+), and effector (CD197−CD45RO−) population. Furthermore, the memory population was divided in two subsets; the CD197+CD45RO+ subset from which the CM (CD28+CD27+) T cells were defined and the CD197−CD45RO+ subset from which the frequency of EMRO (CD28+/−CD27+/−) T cells was determined (A and C). Next in Fig. S2B and D, the gating strategy is shown how we determined the frequency of CD57+ T cells in total CD8+ and CD8− (CD4) T cell populations and the loss of CD28 within the effector population (as gated in A and C). (PDF 544 kb) [file 10875_2016_363_MOESM2_ESM.pdf]

# Supplementary Figure 3

**A**

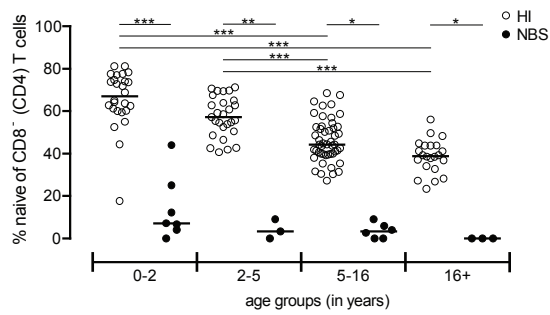

**B**

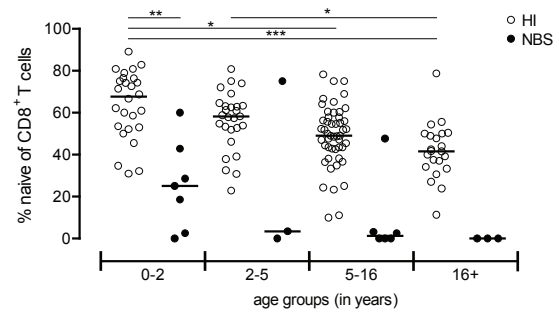

**C**

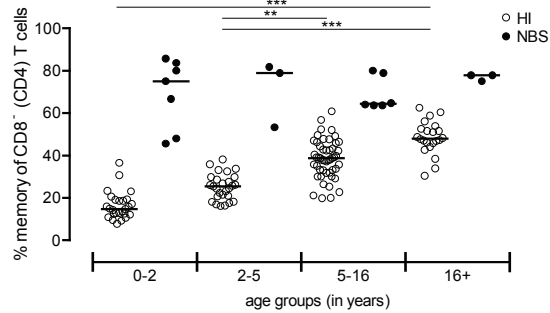

**D**

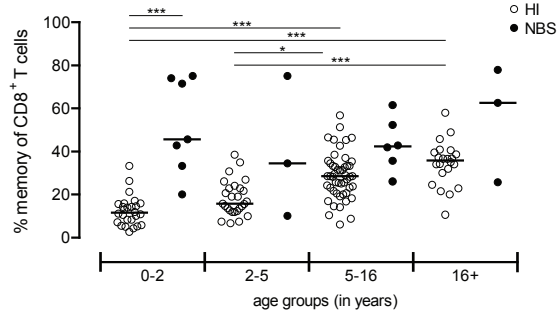

**E**

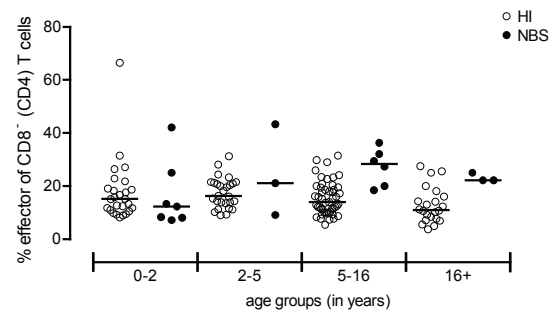

**F**

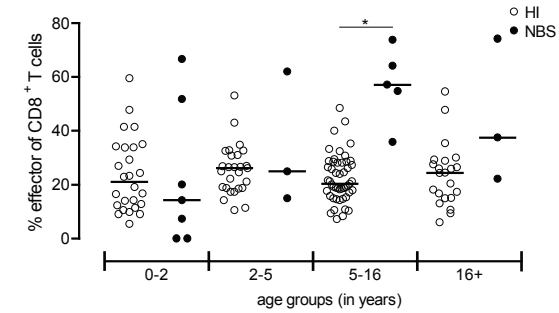

**G**

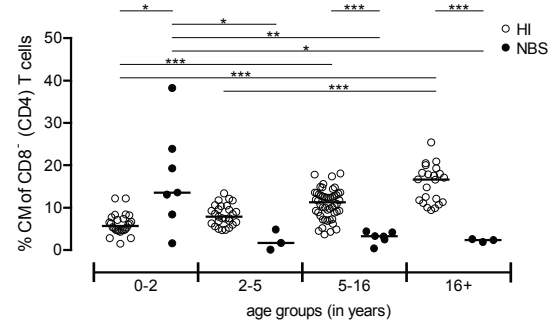

**H**

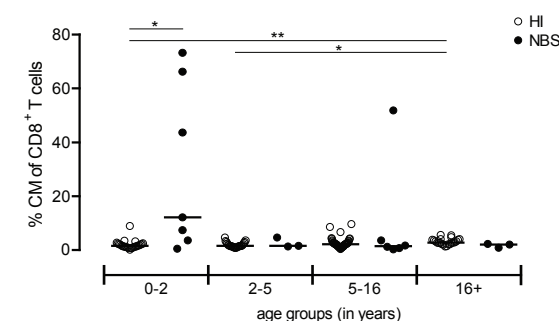

**I**

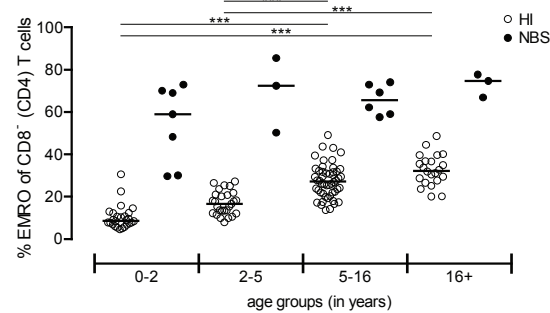

**J**

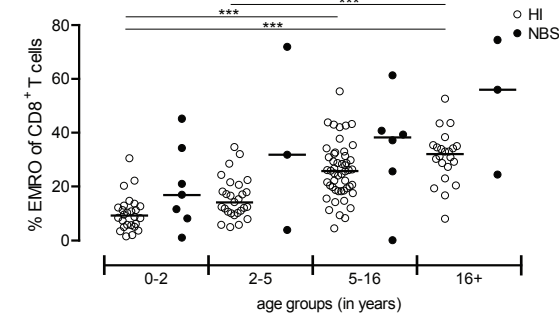

Supplement: Supplementary file 3 — Frequencies of the T cell subsets. Within CD8− (CD4) and CD8+ percentages of naïve (A and B), memory (C and D), effector (E and F), CM (G and H), and EMRO (I and J) are shown of the NBS patients (n = 20, black dots) which were compared to HI (n = 125, open dots). NBS patients and HI were divided on the basis of their age into four groups (age distribution (n = HI vs. NBS), respectively): 0–2 (n = 26 vs. 7), 2–5 (n = 27 vs. 3), 5–16 (n = 50 vs. 6), and 16+ years old (n = 22 vs. 3). Data represents individual measurements and medians. Significant differences between patients and HI and different age groups were shown (* = p < 0.05, ** = p < 0.01, *** = p < 0.001). (PDF 607 kb) [file 10875_2016_363_MOESM3_ESM.pdf]
